# Digestion and Its Relation to the Teeth

**Published:** 1852-10

**Authors:** W. R. Handy


### ARTICLE V.

### *Digestion and its Relation to the Teeth.* By W. R. HANDY, M. D.

DIGESTION, it is well known, constitutes one of the fundamental functions of the human body, and, without detracting from the rest, may, with strict propriety, be regarded as the foundation, or basement story, to all the others; and, of course, by absolute necessity upon its integrity and security, must be involved the integrity and well-being of all the rest.

And if this be true, the question meets us at the very threshhold, viz. How vastly important must this function be in its relative contrast with other functions; and how necessary that a knowledge of this function should first be obtained, as preparatory and indispensable to a proper acquaintance with the others.

Now, if digestion has this fundamental relation to the other great prominent functions, composed of absorption, circulation, secretion, &c., what shall be said of its relation to a part of its own function? or, as strange as may sound the expression, what relation has it, in other words, to a part of itself—as, for instance, the teeth, the subject about to be discussed?

To an intelligent mind, to ask such a question—as whether a part has any relation with the whole, or the whole with any of its parts, seems both absurd and trifling; but, to go further, and attempt a grave discussion of such a matter, would be considered an insult to common sense.

It is, nevertheless, true, that such a discussion not only seems to be necessary, but in our humble opinion, absolutely required in the present state of dental science; for dentistry as a science, though now no longer questioned; her position, notwithstanding, as such, is yet so much in its infancy, that a legion by name, of her pretended votaries, both in deed and word, virtually deny that there is any legitimate or practical relationship between digestion and the teeth, as well as other branches embraced in dental science; and, consequently, such a discussion, if it does no more, will serve, at least, to place all such pretenders in their true position, and of those who are ignorantly honest, to come out (from among them) and properly qualify themselves in the only way they can hope to be respectable in the profession, as well as useful and honest in their daily practice.

That these remarks are not out of place here, we have only to put the question, viz. What is the notion which a mechanical dentist has of this relation between the teeth and digestion? Does he regard the teeth anything more than so many hard, isolated bodies, fixed, he knows not how, into the jaws, but held there, as he must suppose, and what his practice would compel us to believe, just as so many nails driven into a piece of wood, and having just about the same amount of connection and vital endowments?

Without any harshness of language, we think it may, with truth, be said, that the simple mechanical dentist does not even know that digestion belongs to any other part of the body, than the abdominal cavity. And if, perchance, his knowledge should have extended this far, he can still have no conception of what the teeth, so far removed from the "belly," should have to do with digestion; and, hence, what practical utility there is, in his meddling himself to know.

It is true, he will admit that when food is taken into the mouth, that the teeth masticate or grind it up, just as the stones of the grist mill, operate in forming flour; and there, he supposes, is an end of the matter. And scarcely any more conceives that this same food, after a long and complex journey, returns again prepared to build up, beautify, and preserve these same teeth, than he does, that the wheat ground into flour by the stones can (and should, in like manner,) enter into their constitution and preservation.

For the benefit, therefore, of this very large class of mechanical dentists, as well as for the dental student, we propose presenting proof of the undoubted claims of relationship between the teeth and digestion—of this relation being mutual and indispensable;—and, further, that no dentist, without the knowledge of this relation, can practice either with skill or safety to his patient, and, consequently, has no honest, moral, or conscientious right to practice at all.

It will be readily perceived that the subject in hand is a very extensive as well as a most interesting and practical one, as it claims for consideration no less than three great elements, viz. *digestion*, the *teeth*, and the *relation* which these two bear, the one to the other.

The main point, however, in our remarks, will be in directing attention to the *practical relation* of digestion and the teeth.

We shall offer the proof of this relation under the following heads, viz.

1. Anatomical relation of digestion to the teeth.
2. Physiological relation, &c.
3. Pathological relation, &c.
4. Hygienic relation, &c.

Digestion, all are aware, is a complex act—that its function consists of many sub-divisions or subordinate acts, constituting so many minor functions, and that neither one or two, singly, but the whole, collectively, form the function.

Now, to the whole of this function, and each of its several parts, are allotted organs, the especial instruments or agents by which the various operations are carried on; and these organs are styled organs of digestion.

The *grand object* in digestion is, to reduce alimentary matter to a condition in which it may be taken up by absorption and introduced into the system. This object is the same in all the various classes of animals; but the function, nevertheless, (in many of these classes,) presents modifications, requiring a corresponding modification in the various organs—in some, the function is very simple, and the corresponding organs equally simple;—and from this point can be traced, all the different varieties, having each equally as great a diversity in the several organs belonging thereto; and which diversity, (in the digestive process,) says Messrs. Todd & Bowman, occur from "peculiarities in the habits of the animals, or in the nature of their food, and also according to the complexity of organization exhibited by them."

Among many of the inferior animals, the relation between digestion and the teeth is well marked: as, for instance, in the class of *carnivora,* animals that feed on flesh, we find their teeth admirably adapted for seizing and tearing their prey, by which provision this kind of food is fitted for digestion, and thus clearly shows the close and necessary relation which nature has established between this class of teeth, and the digestion of this particular kind of food.

On the other hand, in the class of *herbivora,* whose food consists of vegetables, grain, and fruits, we see the teeth suited to the grinding process, by which arrangement alone, they could be adapted to such kind of food; and thus, shows an equally close relation between the teeth of this class, and the digestion of this peculiar kind of food, upon which they exclusively subsist;* and so in numerous other examples, the relation will be found to be equally close. But our business is chiefly with human digestion and the teeth, to which we wish particularly to confine our remarks.

Human digestion, as already stated, consists of several subdivisions or minor functions, which are called *prehension, mastication, insalivation, deglutition, chymification,* and *chylification.*

The organs corresponding to these several functions are, viz. those of the mouth, the pharynx, œsophagus, stomach, intestines, liver, and pancreas. In other words, the digestive organs comprise the alimentary tube, from the mouth to the anus; at the beginning of which tube we find the teeth, where is also the beginning of digestion; while along its continuous track the digestive function is carried on and completed.

The anatomical relation, therefore, between digestion and the teeth, seems both clear and irresistible, as the latter organs are

* In the carnivora, the stomach is simple and intestines short; while in the herbivora, with more complex dental apparatus, the stomach is also more complex, and intestines much longer, with a cœcum of "great size."

among the first to commence the process. But this is not all, *mucus membrane* constitutes the grand anatomical element, belonging to the function of digestion—it lines the whole of the alimentary tube—is continuous throughout from the mouth to the anus, and is filled with a multitude of glands, connected either directly or indirectly with it, and all of which pour their fluids upon the interior of its surface, which fluids we shall hereafter have occasion to notice, take an essential part in the function of digestion.

Now, this mucous membrane, Mr. Goodsir clearly demonstrates as giving origin to the teeth; thus, showing, with still greater clearness, if possible, the direct anatomical relation of the teeth with digestion. The direct anatomical continuity of those beautiful pearly appendages with that great mucous track, by which digestion is performed. And still further, this same element has the food to come in contact with it at every point; and it is this same food which undergoes digestion, and from which, in the lungs, the blood is formed, and out of which blood, the materials are derived for constructing the various organs of the body; and as the teeth are among these organs, and thus built up from the blood, and the blood thus formed from digestion, the anatomical relation, by the blood-vessels, is also equally established between digestion and the teeth.

Another anatomical element of this relation, we find in the nerves. To the glosso-pharyngeal and gustatory branch of the fifth, are assigned the duty of presiding over and regulating the taste; while other branches of the fifth go directly to the teeth, and belong to general sensibility. Now, this taste, which is placed among the special senses, is our guide in the proper choice of food; is, consequently, connected with, and necessary to digestion; and, as a further consequence from its nervous connection with the teeth, must also place the latter in the same relation with digestion.

The *physiological relation* of digestion with the teeth, is quite as close, if not more so, than the anatomical. Here, the relation is in its natural healthy state. To the teeth is assigned the function of mastication, which is simply one of the acts, among the numerous others, which, in the aggregate, constitute the great function of digestion. In other words, these various acts, as the several links of a chain, constitute one undivided and necessary whole—so much so, that in the language of the poet:

> "Whatever link you strike,
> Tenth, or ten-thousandth, breaks the chain alike."

Which is to say, that however inferior mastication may seem, in contrast with chymification, or chylification, in the function of digestion, it is, nevertheless, an important link in the chain, which, if you strike out or disturb, will, in a proportionate degree, interrupt or disturb the healthy relations of this process.

This brings us to the *pathological relation* of digestion with the teeth, where we find this function or the teeth, or both, in a state of disease; where the harmony, in one or more of the links of the great digestive process is disturbed; and, of course, where the dependency among the several parts must be equally disturbed, and where, hence, also, the relation between each must likewise be equally affected.

*Dyspepsia* is regarded as a disease most especially invading the healthy process of digestion. The mucous membrane of the stomach is the anatomical element, most particularly assigned as its seat; while the influence of its morbid power is most wofully felt over the entire man, in depressing all his physical as well as mental energies. The nerves are the especial agents in radiating this morbid influence from the stomach to every part of the body; and the par-vagum of the eighth pair seems to take the most prominent part in carrying the disease, as well as reporting its existence and severity to the great centres of nervous action; for this nerve, whose numerous branches may be traced over the stomach, can be distinctly followed upwards through the diaphragm, supplying this latter organ, and thence ascending the posterior mediastinum upon the œsophagus, which being also supplied, it then sends off, in its course, branches to the lungs and the heart, and still pursuing an upward course through the neck, we find it supplying here, the trachea and the larynx, besides entering into the formation of plexuses; and yet ascending, we trace it finally to its source, the medulla-oblongata, the great cerebro-spinal centre, the bond of union between the brain and spinal-marrow, and the especial source of nervous control over the vital function of respiration. We thus see this single nerve connecting the stomach, diaphragm, œsophagus, lungs, heart, trachea, larynx, spinal-marrow, and brain, organs most important, both to the existence, as well as maintenance of the being; for these are organs, which, all are aware, hold the very keys of life and death, as they involve the functions of respiration, circulation, digestion, and the intellect —functions by which we breathe, by which our food is prepared and distributed, and by which the mental energy of the man is displayed. And, hence, dyspepsia, the very essence of indigestion, scattering such devastation over the entire man, both body and mind, is it possible for the dental apparatus to escape its destructive march? Can we, for a moment, believe? Ay, may we not go further, and ask, if it be possible for even a mechanical dentist to believe that amidst this general havoc, the teeth alone escape, and that they do so, simply by some fancied independence of the rest of the body, in defiance of the relation which nature has declared, most positively, to exist between the whole body and its several parts, and, also, as to whether that body be in health or disease? For in both conditions, she has equally declared the relation inseparable.

But as this disordered digestion by dyspepsia upon the teeth, will have an important bearing upon the question, as to the right of a dentist to practice, who is ignorant of this relation, it seems necessary that we should enter into some further explanation, as to how this diseased process of indigestion upon the teeth is so brought about as to unequivocally establish the relation between the two.

If there be any truth in the remarks already made, the explanation cannot seem very difficult; for, as already stated, the function of digestion, as well as the instruments of its performance, are in an abnormal condition—the condition of disease—a complete departure both from healthy structure, as well as healthy function.

Now, it is not only inconceivable, but, further, in direct contradiction of the best established principles both in physiology and pathology, that any organ can have a healthy foundation and superstructure, when the very materials entering into the formation of such organ are in a state of disease.

It has been already shown, that our element of food and drinks is converted into chyme and chyle, and that from this latter the blood is formed, and that from the blood, is derived both the building materials of every organ, as well as the source of its continued existence and future sustentation; and the teeth, it was asserted, formed no exception to this general law, for they have the same essential elements constituting those organs, as any other organ in the body. For instance, they are obedient to the same organic laws, by having a birth, growth, development, decay, and death, as that of any other organ, or as the general body itself. They possess, also, an equally determinate form, size, and limited duration; are, also, equally endowed with the same essential anatomical elements, viz. blood-vessels and nerves, and equally vitalized and nourished by the same indispensable fluid, the blood; and as it is from the blood that the various elements for constructing the different organs, with the teeth, are derived, it is only necessary for us to show that the chyme and chyle, formed by the function of digestion, and from which the blood is derived, is at fault—to show that the blood also is at fault—and, consequently, the teeth, formed from the blood, must, also, be equally at fault; in other words, having an imperfect construction, which is either disease developed, or a predisposition strongly tending thereto.

Now, that we should expect healthy chyme and chyle to be formed in the stomach and duodenum, when the gastric juice and the bile and pancreatic fluids are wanting or deficient in quantity or quality, and this, too, to take place in organs, which, themselves, are in a state of disease, and crippled in all their healthy actions, we say, to look for such a result seems about as preposterous as to expect that "grapes should be gathered of thorns, or figs of thistles." But it may be said, that the quality of the food will make up the difference as to purity in the formation of the chyme and chyle, the answer still is, that no matter how good the food may be, still, the organs cannot digest it; or, if the function be carried on at all, digest it but very imperfectly, and, consequently, very bad chyme and chyle, or none at all is formed, and just as certainly must there be a like character to the after blood; and as a still further result, must all the after organs be, which are formed from it, viz. partaking alike in the imperfection, and, consequently, necessarily stamped with a departure from the natural standard of health, which departure the scientific dentist has an opportunity of witnessing with greater distinctness and accuracy, than the physician, for with him the teeth are exposed to more exact observation during a considerable period of their growth; so that, to his experienced eye and enlightened mind, the different shades of color, and different degrees of density, which, if wanting in any of the elements necessary to constitute sound and healthy teeth, point him with quick perception, and almost infallible certainty, first, to bad blood, then to bad chyle, bad digestion, bad health, and, as a necessary consequence, also bad teeth.

The direct pathological relation, therefore, of digestion with the teeth seems sufficiently conclusive, from this single example of dyspepsia, alone, without referring to any others; so, that we shall pass on to the last head under which we proposed to consider this relation, and that is, viz.

The *hygienic relation* of digestion with the teeth.

This relation has reference to the rigid application of all the laws of health, as the best preservative from disease of both digestion and the teeth; or, if digestion be impaired, and as, is often the case, this impairment depends on dental disease, by observing the hygienic laws, in having the teeth in a state of health, will go very far to restore, if not entirely remedy, the fault in digestion. On the other hand, if the teeth be diseased during their formation, a sound digestion will arrest this, and restore them to a healthy state. These hygienic laws refer to both sets of teeth, temporary, as well as permanent, and in every stage of their existence, as well as to the functions of digestion from its earliest dawn to its latest moment.

Now, if all this be true, how can any mechanical dentist, who is entirely ignorant of these laws, instruct a mother, when she may be enciente in reference to her teeth? their influence upon her digestion? and the certainty of this influence upon the temporary teeth of her offspring? As he knows nothing of the relation of the fetus in utero to the digestion and bad teeth of the mother, it seems very clear he is not competent to give any instructions whatever, and by necessary consequence, therefore, has no moral right, whatever, to practice dentistry; and as he has no knowledge that the condition of the blood, during gestation, and the condition of the milk after birth, both exert a most decided and positive influence, either for weal or woe, upon the fetal, as well as the after periods of the teeth of the child. It is, also, equally clear, that he has no right to practice the dental profession. To constitute, therefore, the right to practice this high and humane calling, we think we are justified in safely asserting that it belongs of right—we mean of moral conscientious right—to none but the scientific dentist, the man who acknowledges, studies, and applies all the great fundamental principles and facts of medical science, as necessary to the skillful, as well as the successful practice in the particular specialty of his choice, viz. the dental profession.

In other words, he regards it as necessary in dental, as well as medical science, that every dentist, equally with the physician, should build his foundation upon anatomy and physiology, and have his superstructure reared by the lights of pathology, practice, and therapeutics, that the details of his dental practice may thus stand out in bold relief, by being strengthened with all the improvements of science, as well as presenting the highest claims of legitimate qualification to the community; and thus offering the greatest security which this community can have, in its time of dental suffering, by being provided with safe and skillful hands: and, in being so provided, it follows, as a still further consequence, that every such community should cheerfully and liberally patronize all such dentists, and, equally, with the scientific physician, regard them both as the guardians of their health, as well as the ministers to their comfort.

Having now discussed as far as we deem necessary, or, perhaps, profitable, the relation between digestion and the teeth, and, perhaps, also, of having satisfied some of our mechanical dental friends that they are in the wrong, and are doing violence to a profession, to which they have unlawfully attached themselves—great injustice to a suffering community, on whom they presume to practice—and an undoubted wrong to their owu consciences, which must certainly condemn them, if they have any. We say, under such circumstances of conviction, it may be thought necessary and profitable to all such inquirers after truth and knowledge, that we should enter more largely into the subject of digestion, and give, in detail, the whole anatomy and physiology of this interesting function.

We reply, that our remarks are now more extended than is desirable for the brief article at first intended; and that, still further, such an understanding is entirely unnecessary, as all of our standard works upon anatomy and physiology, not only embrace this whole subject, but much more of equal interest and importance, to which we would respectfully refer the inquirer. Satisfied, ourself, if at all successful, in awaking the great mass of the dental profession to a serious consideration of this whole subject.

